# Environmental Risk Factors in Psoriasis: The Point of View of the Nutritionist

**DOI:** 10.3390/ijerph13070743

**Published:** 2016-07-22

**Authors:** Luigi Barrea, Francesca Nappi, Carolina Di Somma, Maria Cristina Savanelli, Andrea Falco, Anna Balato, Nicola Balato, Silvia Savastano

**Affiliations:** 1 I.O.S. & COLEMAN Srl, 80011 Naples, Italy; dott.ssa.nappi@gmail.com (F.N.); cristysav@hotmail.com (M.C.S.); falco.and@gmail.com (A.F.); 2 IRCCS SDN, Napoli Via Gianturco 113, 80143 Naples, Italy; cdisomma@unina.it; 3 Dipartimento di Medicina Clinica e Chirurgia, Unit of Dermatology, Federico II University Medical School of Naples, Via Sergio Pansini 5, 80131 Naples, Italy; anna.balato@unina.it (A.B.); nicola.balato@unina.it (N.B.); 4 Dipartimento di Medicina Clinica e Chirurgia, Unit of Endocrinology, Federico II University Medical School of Naples, Via Sergio Pansini 5, 80131 Naples, Italy; sisavast@unina.it

**Keywords:** environmental risk factors, nutritionist, obesity, body composition, bioelectrical impedance analysis, phase angle, lifestyle, nutrition, Mediterranean diet

## Abstract

Psoriasis is a common, chronic, immune-mediated skin disease with systemic pro-inflammatory activation, where both environmental and genetic factors contribute to its pathogenesis. Among the risk factors for psoriasis, evidence is accumulating that nutrition plays a major role, per se, in psoriasis pathogenesis. In particular, body weight, nutrition, and diet may exacerbate the clinical manifestations, or even trigger the disease. Understanding the epidemiological relationship between obesity and psoriasis is also important for delineating the risk profile for the obesity-related comorbidities commonly found among psoriatic patients. Moreover, obesity can affect both drug’s pharmacokinetics and pharmacodynamics. Additionally, the overall beneficial effects on the obesity-associated comorbidities, clinical recommendations to reduce weight and to adopt a healthy lifestyle could improve the psoriasis severity, particularly in those patients with moderate to severe disease, thus exerting additional therapeutic effects in the conventional treatment in obese patients with psoriasis. Education regarding modifiable environmental factors is essential in the treatment of this disease and represents one of the primary interventions that can affect the prognosis of patients with psoriasis. The goal is to make psoriatic patients and health care providers aware of beneficial dietary interventions. The aim of this review is to assess the relevance of the environmental factors as modifiable risk factors in psoriasis pathogenesis, with particular regard to the involvement of obesity and nutrition in the management of psoriasis, providing also specific nutrition recommendations.

## 1. Introduction

Psoriasis is a common, chronic, immune-mediated skin disease with systemic pro-inflammatory activation, where both environmental and genetic factors contribute to its pathogenesis [1]. Psoriasis usually occurs in the second-to-fourth decade of life, and males and females are equally affected [2].

The worldwide prevalence of psoriasis is around 2%–4% [3], but studies in developed countries have reported higher prevalence rates of on average about 4.6% [4]. Nearly two thirds of patients with psoriasis have a mild form of the disease, with less than 3% of the skin surface of the body affected, but others have more extensive involvement of the skin. Of interest, psoriasis is associated with an increased metabolic risk in a manner that varies with the severity of psoriasis [5,6]. According to recent literature data, psoriatic patients show a greater prevalence of obesity [7] and metabolic syndrome [8], which confers a higher cardiovascular risk [9]. In this, inflammation could be the common link between psoriasis and obesity [7]. In addition, there is growing recognition that psoriasis has a negative impact on quality of life, reduces productivity at work, increases physical disability, and impairs social functioning [10]. Thus, the treatment of environmental modifiable risk factors (i.e., diet, nutrition, or physical activity) and modulation of the systemic inflammatory response are important therapeutic goals in the integrated management of psoriatic patients [11].

In most of the studies evaluating the metabolic risk in psoriatic patients, body mass index (BMI) has been employed as a measure of obesity. BMI is not, however, a measurement of body adiposity as it does not provide a good indication of the ectopic location of fat deposition, mainly visceral adiposity, which has been found to be more closely related to cardiovascular health risk than total fat mass [12,13]. Bioelectrical impedance analysis (BIA) is relatively simple, quick, and non-invasive, which gives reliable measurements of body composition with minimal intra- and inter-observer variability [14]. The results are available immediately and reproducible, with <1% error on repeated measurements [15]. In this contest, only few studies have investigated the association between psoriasis and body composition [16], or have based the specific nutritional assessment on body composition. Recent clinical evidence supports that nutritional assessment may provide a viable support in psoriatic patients to improve either the disease severity and the obesity-related comorbidities [6,17].

Thus, the aim of this review is to highlight the possible relationship between psoriasis and body weight, going beyond BMI, focusing on the impact of obesity on the medical treatment of psoriasis, and the role of diet and nutrition in psoriasis.

## 2. Obesity and Psoriasis

### 2.1. Role of Low-Grade Inflammation

Obesity is an important risk factor for psoriasis [7]. The relationship between the two conditions is probably bidirectional, with obesity predisposing to psoriasis and psoriasis favouring obesity [18]. Obesity is considered a chronic, low-grade inflammatory condition and the adipose tissue is an active endocrine organ that has a key role in lipid and glucose metabolism, inflammation and coagulation, and insulin-mediated processes. Macrophages are the key immune cell type that perpetuates inflammation within adipose tissue. Activated macrophages in adipose tissue stimulate adipocytes to secrete inflammatory mediators that establish and maintain the low-grade inflammatory state in obesity. Adipose tissue, especially visceral adipose tissue, then secretes bioactive products collectively known as adipocytokines or adipokines [19].

Several lines of basic and translational researches suggest that adipocytes and inflammatory-type macrophages are involved in the link between psoriasis and obesity [18]. The co-existence of psoriasis and obesity is at least in part attributed to the function of adipokines and their downstream effects. The role of the various adipokines in psoriasis is an area of active investigation [18]. For example, leptin is an adipokine that serves as an afferent signal of the nutritional and fat mass status to the hypothalamus and thereby regulates appetite and body weight [20]. In addition to the regulation of food intake, leptin plays important roles in the chronic pro-inflammatory state associated with visceral obesity, metabolic syndrome, and their complications, such as atherosclerosis [21], possibly by impairment of insulin signalling at the insulin receptor substrate-1 phosphorylation [22]. Studies in psoriasis have shown that leptin levels are elevated in psoriatic patients compared with healthy controls, and psoriasis is an independent risk factor for hyperleptinemia [23]. Contrariwise, plasma levels of adiponectin, a cytokine with insulin-sensitizing and anti-inflammatory, are decreased in psoriasis patients compared with healthy controls and inversely correlated with psoriasis severity and tumour necrosis factor (TNF)-α levels, the cytokine that has been proposed as a link between obesity and insulin resistance [24]. On the one hand, TNF-α is overexpressed in adipose tissue from obese animals and humans, while on the other hand its excessive production is evidenced in the skin or joints in psoriasis and psoriatic arthritis leading to the rapid growth of skin cells and/or damage to joint tissue. Consequently, TNF-α may also contribute to the extent of psoriatic lesions in obese patients. Blocking the TNF-α production helps stop the inflammatory cycle of psoriatic disease, but it does not improve insulin sensitivity in obese patients with type 2 diabetes [25]. Further evidence of a link between obesity, inflammation, and cardiovascular diseases in patients with psoriasis is provided by several studies reporting a positive correlation between obesity, the clinical severity of disease, evaluated by the Psoriasis Area and Severity Index (PASI) [26], and increased of C-reactive protein (CRP) levels [27], an acute phase protein representing the most sensitive markers of inflammation and an independent risk for cardiovascular disease.

Although obesity likely predates or co-exists with psoriasis, a slightly increased risk for developing obesity has been reported in patients with existing psoriasis compared with controls [7]. Sedentary lifestyle and psoriasis can also be closely connected [28]. Indeed, according to the 2003–2004 and 2005–2006 NHANES dermatology questionnaires [29], psoriatic patients are reluctant to engage in physical activities where their skin disease may be visible to others. Thus, in addition to genetic and immune-mediated mechanisms, behavioural factors may have an additional role in explaining the association between obesity and psoriasis.

As discussed later, BMI greater than 30 kg/m^2^ may potentially play a role in patients’ abilities to achieve the full therapeutic effect of psoriasis therapy. This could be for two possible reasons: it may be a consequence of decreased drug distribution into the body, as a result of increased body mass, or it may be a consequence of increased pro-inflammatory cytokine release, as a result of the adipocyte dysfunction [7,18,19]. Given the potential correlation between elevated BMI and increasing psoriasis severity, weight loss has been considered as a potential adjunct to other psoriasis therapies. Romero-Talamás and colleagues [30] reported that almost 40% of 33 morbidly obese individuals with psoriasis on active medical treatment undergoing bariatric surgery showed significant improvement of clinical severity of psoriasis positively related to the degree of postoperative weight loss.

### 2.2. Obesity: Going beyond the BMI

A diagnosis of obesity is established by determining the patient’s BMI using the formula weight in kilograms divided by the square of the height in meters. The current World Health Organization weight classification for BMI in adults is as follows: a BMI of between 18.5 kg/m^2^ and 24.9 kg/m^2^ is normal, 25.0 kg/m^2^ to 29.9 kg/m^2^ is overweight, and a BMI greater than 30.0 kg/m^2^ is diagnostic of obesity [31].

Several studies have been conducted to explore the association between obesity and psoriasis using BMI [32,33]. However, the epidemiological evidence currently available is insufficient to establish which comes first, obesity or psoriasis [18]. In particular, there was a two-fold increased risk for psoriasis development in the setting of obesity as compared with normal weight subjects [34]. In addition, for each unit increment increase in BMI was reported a 9% higher risk for psoriasis onset and a 7% higher risk for increased of PASI score [35]. BMI is a commonly used surrogate for adiposity that is inexpensive and easily measured. Nevertheless, BMI evaluates excess weight rather than excess fat [36], as it does not measure body fat directly, and poorly distinguishes between fat mass and lean or bone mass [37]. The National Heart, Lung and Blood Institute Clinical Guidelines recognizes this limitation of BMI [38]; thus, waist circumference (WC) is recommended as an additional surrogate measure of fat distribution, due to its high correlation with visceral fat [39], the main source of inflammatory cytokines in obesity [40]. While in most of the studies evaluating the association between obesity and psoriasis, BMI has been employed as a measure of obesity, a number of studies have evidenced a strict association between WC and psoriasis [41], being the production of inflammatory cytokines in visceral obesity the link involved in the complex mechanisms leading to the exacerbation of psoriasis [16]. Only few studies have assessed body composition in psoriatic patients by BIA [42,43,44]. Significant relationships have been evidenced between body composition and the occurrence of psoriasis [45], the severity of the disease [46], or the response to treatment with anti-TNF-α agents [47]. However, BIA is not a direct method for assessment of body composition and its accuracy as an indicator of body composition could be hampered by an altered distribution of extra- and intra-cellular water [48].

Differently from the other parameters obtained by BIA, the phase angle (PhA), a direct BIA measure, is a rapid, easy, and bloodless tool in clinical setting and is a general indicator of cell membrane integrity. The PhA represents either the reactance of tissues associated with cellularity, cell size and integrity of the cell membrane, and the resistance of tissues, which is dependent on lean tissue mass and tissue hydration [48]. It is well established that a decrease in PhA is consistent with cell death, reflecting a breakdown of cell membranes. Consequently, the loss of the cell membrane integrity results in a decrease in intracellular water, an increased spacing among affected cells, with the expansion of the interstitial fluid space. On the other hand, larger PhAs reflect higher quantities of intact cell membranes and lean body mass [49].

Very recently, we have reported a novel association between PhA, and psoriasis. In particular, our data well demonstrated that PhA was smaller in psoriatic patients than in healthy subjects, and that this difference is independent of gender. In addition, we found significant correlations between PhA with the clinical severity of psoriasis, expressed by PASI score, CRP levels, and the quality of life in these patients, independently of BMI. Based on ROC curve analysis, PhAs ≤4.8° and ≤4.9° identified psoriatic patients who have the highest clinical severity and the lowest quality of life, respectively [50].

### 2.3. Impact of Obesity on the Treatment of Psoriasis

The cornerstones of treatment of the obesity are the management of weight and ensuring appropriate levels of physical activity, as demonstrated by studies showing that dietary modification and enhanced physical activity may delay or prevent the transition from impaired glucose tolerance to type-2 diabetes [51]. Obesity also has important implications in the treatment of psoriasis. Indeed, obesity has been associated with a decreased response to systemic and biologic therapies [18]. This may be due to pharmacokinetic factors and affect more particularly the drugs administered in fixed doses than those in which dose is adjusted to the patient’s weight. On the one hand, obese patients with psoriasis are at greater risk of adverse effects with conventional systemic drugs. In particular, those biological drugs in which dose is not weight-adjusted, such as etanercept and adalimumab, may be less effective, where other biological drugs, such as infliximab and ustekinumab, for which dose is weight-adjusted, the increased cost of treatment should also be considered [18]. Weight loss will decrease the risk of drug toxicity and enhance effectiveness and tolerance, particularly in the case of drugs administered at fixed doses. In addition to the potential decrease in the effectiveness of treatment and the greater risk of adverse effects, obesity also substantially increases the cost of treatment with drugs prescribed in weight-adjusted doses. Bardazzi and colleagues reported that in 33 moderate to severe psoriasis patients treated with biological drugs, the only seven patients who decreased their weight achieved the highest improvement in mean PASI score at the end of the study, even when not responding at the first follow up [52]. More recently, evidence has been provided that in obese patients with psoriasis on biologics undergoing a 8 weeks dietary programme with a low calorie diet (≤1000 kcal per day), the body weight reduction was associated with an increase in the efficacy of the drug [53]. Therefore, the lifestyle interventions, particularly weight reduction, may even enhance the efficacy of psoriasis treatment, as demonstrated in a recent randomized trial and by case reports of dramatic improvement of psoriasis after bariatric surgery in morbidly obese psoriatic patients [18]. A further aim of the management of obese patients with psoriasis should be to achieve also a reduction in the obesity-related inflammation. On the other hand, obesity is associated with conditions such as metabolic syndrome and hepatic steatosis, which can increase the risk of adverse effects to conventional systemic treatment for psoriasis [6].

## 3. Nutrition and Diet in Psoriasis

Among the risk factors for psoriasis, evidence is accumulating that nutrition plays a major role, *per se*, either in the pathogenesis of psoriasis or in affecting drug pharmacokinetics and pharmacodynamics [18,54,55]. However, in the vast majority of the results it is difficult to discriminate between the effect of weight loss and dieting *per se*.

Severe psoriasis has been associated with nutritional deficiencies because of an accelerated loss of nutrients from the hyperproliferation and desquamation of the epidermal layer of skin [56]. Indeed, in severe cases the psoriasis can result in an insufficient nutritional status which may even be promoted by nutrient-drug interactions. Limited data exist regarding the role of specific diet regimens in psoriasis, mainly with the aim to reduce cardiac risk factors and obesity-related comorbidities. Previous studies or single case reports reported the positive effects of low-energy diets and vegetarian diets [54], formula diet weight loss programmes [57], gluten-free diet [58], very low-calorie carbohydrate-free (ketogenic) [59]. Fasting periods or vegetarian diets [54,55], and diets rich in omega-3 polyunsaturated fatty acids (ω-3 PUFA) from fish oil [60] have been associated with improvement of psoriasis in clinical trials. In this, the reduced amounts of arachidonic acid and the increased eicosapentaenoic acid intake might result in an anti-inflammatory environment [61]. Some psoriatic patients are gluten-sensitive and may benefit from a gluten-free diet [58,59,62]. It is believed that some vitamins (A, E and C), and oligoelements (iron, copper, manganese, zinc, and selenium) have anti-oxidants ability, which decrease oxidative stress and the production of reactive oxygen species [17,63]. In addition, along with improving glucose, insulin and lipid control, food fibres also play an important role in systemic inflammation, by decreasing the oxidative stress produced by the elevated intake of high-simple carbohydrate foods. Finally, due to its role in proliferation and maturation of keratinocytes, vitamin D has become an important therapeutic option in the treatment of psoriasis [64].

Monounsaturated fatty acids (MUFA) are considered a healthy dietary fat, as opposed to saturated fatty acid. The most frequently consumed MUFA rich dietary oils is extra virgin olive oil (EVOO). Traditionally, the beneficial effects of EVOO have been attributed to its high MUFA content (oleic acid), as it protects lipoproteins and cellular membranes from oxidative damage [65]. Very recently, we have demonstrated that psoriatic patients, compared to the control group, have a higher consumption of simple carbohydrates, total fat and ω-6/ω-3 PUFA ratio, with a lower intake of protein, complex carbohydrates, MUFA, ω-3 PUFA, and fibres [66]. In particular, in this study we found that the lowest intake of MUFA was associated with the highest clinical severity of psoriasis. The association between low MUFA intake and progression of psoriasis is in line with the same observation reported in other chronic inflammatory diseases [67,68]. Additionally, the relationship of psoriasis with either individual nutrients or individual food groups, it should be kept in mind that diet is a complex combination of foods from various groups and nutrients, and some nutrients are highly correlated. Thus, it is challenging to separate the effect of a single nutrient or food group from that of others in free-living populations [69].

## 4. Association between the Severity of the Disease and Adherence to the Mediterranean Diet

Recent evidence has confirmed that adherence to a healthy diet over time reduces the risk of long-term inflammation [70,71,72]. In particular, Barbaresko et al. [72] reported that fruit and vegetable-based healthy dietary patterns were associated with lower biomarkers of inflammation, such as CRP levels.

The traditional Mediterranean diet (MD) is a healthy diet characterised with the abundance of vegetable foods and cereals, such as green and yellow vegetables, salads, legumes, bread, pasta, fruits and nuts [73]. MD is a highly palatable and favourable diet and may lead to a higher adherence among dieters in the long term. EVOO is the main source of fat and the intake of fish, poultry, dairy products, and eggs is moderate. In addition, different amounts of wine are usually consumed in moderation with meals. Animal fats used in butter, cream, and lard are not included in this diet. The MD is considered a healthy eating pattern, associated with reduced risk for metabolic [74], cardiovascular [75], neoplastic [76], and chronic inflammatory diseases [77]. One of the most accredited hypothesis of this association is that the high content of different beneficial compounds, such as antioxidants and polyphenols, largely present in Mediterranean foods, such as plant foods, fruits and red wine, have anti-inflammatory and antioxidant properties [60,61,78]. In particular, the MUFA intake have health benefits on the reduction of a coronary heart disease risk, the prevention of several types of cancers, the modification of the immune and inflammatory responses and the reduction of the osteoporosis risk [64,65,79]. The association between MD and the lowered incidence of chronic inflammatory diseases is well-supported by intervention studies with the MD [80].

Although the exact mechanisms of these protective properties are not fully unravelled, β-carotenoids, folic acid, and fibres, characteristic for this diet, are known to play a pivotal role in the prevention of oxidative stress [17]. Different studies evidenced that single foods typical of MD, such as fruits, vegetables, and whole grains, are associated with lower concentrations of CRP levels [81,82]. In contrast, following intake of energy-dense, nutrient-poor, processed foods, meal-induced inflammation has been evidenced by immediate increases in CRP levels [83]. In this, we have recently reported that, among the basic components of the MD, the intake of EVOO and fish have an independent predictive value for clinical severity and CRP levels in psoriatic patients [46]. In addition, other nutrient and non-nutrient components of MD foods, such as β-carotene, zinc, selenium, vitamin C, and vitamin E, have been shown to be associated with lower levels of markers of inflammation [84]. In particular, the flavonoid compounds present in vegetables, the most important sources of phenolic, are considered to mainly provide the antioxidant effects [58]. In addition, vegetables are also an important source of phytosterols that reduce cholesterol serum levels and, subsequently, the cardiovascular risk [63]. Protective actions against oxidative mechanisms are also exerted by fruits, other basic element of the MD, due to the high amount of fibres, vitamins, minerals, flavonoids, and terpenes. Even condiments commonly used in the MD to increase the palatability, including garlic, onions, cappers, herbs, contain large quantities of flavonoids or allicin, the latter known to have cardiovascular and neurocognitive benefits [62,75]. The ω-3 PUFA, (eicosapentaenoic and docosahexaenoic acids), mainly found in fish and nuts, contribute to provide the protection of several chronic diseases [64]. EVOO, besides the high levels of MUFA, is a source of several phytochemicals (i.e., polyphenolic compounds, squalene and α-tocopherol). Finally, the dairy products of the MD, such as yoghurt, are better tolerated by the lactose-intolerant subjects and might induce favourable changes in the gut microflora, with positive effect also on the risk of colon cancer [74]. 

In summary, rich diversity of foods of the traditional MD encompasses these dietary characteristics resulting in a unique compendium of nutrients that contributes to its protective effects against psoriasis and other chronic inflammatory diseases. 

Recent data provided by our group demonstrated that there was a significant association the adherence to the MD with the severity of psoriasis, and CRP levels. In particular, the results of our study showed that a higher percentage of psoriatic patients have a low adherence to the MD compared with the age- sex- and BMI-matched control group, with a strict relationship between a higher consumption of EVOO and a lower psoriasis severity. This association suggests that the beneficial effects of nutritional interventions promoting the Mediterranean food pattern could be extended to psoriatic patients. 

## 5. The Point of View of the Nutritionist

The nutritional assessment, based on body composition, and lifestyle modifications should be an integral component of management of the psoriatic patients. These “easy” concepts might be of strategic relevance in terms of clinical efficacy and cost-effectiveness of the newer biological drugs. A diet regimen rich in MUFA and ω-3 PUFA, fruits, vegetables, fibre, with the reduced intake of saturated fats, simple carbohydrates, and sweetened drinks, should be recommended to the psoriatic patients. The role of nutritional supplements has been extensively evaluated in a recent review [17]. Nutrition and dietetic recommendations might be summarized as follows:

Foods to include:

- Cold water fish, such as salmon, herring, mackerel, and trout, due to their content in ω-3 PUFA and vitamin D. It is recommended especially to prefer wild fish (not farmed), to also prevent the ingestion of harmful chemicals and toxic additives. Flaxseed and evening primrose oil might be also used as additional dietary sources.
- Whole grains, legumes, vegetables, and fruits as rich source dietary fibre, trace elements, including zinc and selenium, and vitamins. In particular, orange and yellow vegetables contain vitamin A, pumpkin seeds that provide zinc, often deficient in patients with psoriasis [85], and brewer’s yeast, as its content in vitamin B_12_ has been reported to be effective in improving the psoriatic skin lesion [17].
- EVOO, as main source of dietary fat.
- Foods to avoid: Alcohol, gluten, given the higher prevalence of celiac disease among psoriatic patients, red meat, and dairy products, due to their high content in saturated fat, peppers; the association between caffeine, including coffee, tea black, mate, dark chocolate, remains still to be ascertained [86].
- In addition to fish oil rich in ω-3 PUFA, other dietary supplements with vitamin D and B_12_ or selenium still need further evidence on their clinical effectiveness in large population samples.

The goal of these recommendations is to make psoriatic patients and health care providers aware of beneficial dietary interventions. Achieving dietary-related goals includes an integrated effort of a trained team that involves the psoriatic patients in the decision-making process. It should be recommended that, among the team members a leading role in providing nutrition care should be given to skilled nutritionists, with all team members knowledgeable about the dietary interventions and supporting nutrition and dietetic recommendations.

## 6. Conclusions

Significant associations between environmental risk factors and psoriasis have been systematically observed. Understanding the bidirectional relationships between obesity and psoriasis is also important for delineating the risk profile for comorbidities that may result from obesity. Weight loss might be of strategic relevance in terms of the clinical efficacy and the cost-effectiveness of the newer biological drugs for treatment of psoriasis. In addition to weight loss, to adopt a healthy lifestyle could have per se beneficial effects on psoriasis severity, particularly in those with moderate to severe disease, and exert additional therapeutic effects in the conventional treatment in obese patients with psoriasis. Education regarding modifiable environmental factors (diet, nutrition, appropriate weight, and physical activity) is essential in the treatment of this disease and represents one of the primary interventions that can affect the prognosis of patients with psoriasis. Achieving dietary-related goals includes an integrated effort of a trained team, where skilled nutritionists should play a central role and the psoriatic patients should actively participate in the decision-making process. On the basis of its components, the MD presents health benefits and can prevent obesity, diabetes, and cardiovascular conditions. The beneficial effects of nutritional interventions promoting the Mediterranean food pattern could be extended to psoriatic patients. Future well-designed dietary intervention trials on larger population samples are needed to define specific dietary guidelines for psoriasis.
